# Go To Travel campaign and the geographic spread of COVID-19 in Japan

**DOI:** 10.1186/s12879-022-07799-0

**Published:** 2022-10-31

**Authors:** Asami Anzai, Sung-mok Jung, Hiroshi Nishiura

**Affiliations:** Graduate School of Medicine, Kyoto University, Yoshidakonoecho, Sakyo-Ku, Kyoto, 606-8501 Japan

**Keywords:** Tourism, Mobility, Spatial analysis, Causal inference, Economic campaign, Time-series data

## Abstract

**Background:**

In 2020, the Japanese government implemented first of two Go To Travel campaigns to promote the tourism sector as well as eating and drinking establishments, especially in remote areas. The present study aimed to explore the relationship between enhanced travel and geographic propagation of COVID-19 across Japan, focusing on the second campaign with nationwide large-scale economic boost in 2020.

**Methods:**

We carried out an interrupted time-series analysis to identify the possible cause-outcome relationship between the Go To Travel campaign and the spread of infection to nonurban areas in Japan. Specifically, we counted the number of prefectures that experienced a weekly incidence of three, five, and seven COVID-19 cases or more per 100,000 population, and we compared the rate of change before and after the campaign.

**Results:**

Three threshold values and three different models identified an increasing number of prefectures above the threshold, indicating that the inter-prefectural spread intensified following the launch of the second Go To Travel campaign from October 1st, 2020. The simplest model that accounted for an increase in the rate of change only provided the best fit. We estimated that 0.24 (95% confidence interval 0.15 to 0.34) additional prefectures newly exceeded five COVID-19 cases per 100,000 population per week during the second campaign.

**Conclusions:**

The enhanced movement resulting from the Go To Travel campaign facilitated spatial spread of COVID-19 from urban to nonurban locations, where health-care capacity may have been limited.

**Supplementary Information:**

The online version contains supplementary material available at 10.1186/s12879-022-07799-0.

## Background

Since the first case of severe acute respiratory syndrome coronavirus 2 (SARS-CoV-2) infection was reported in January 2020, Japan had experienced seven waves of coronavirus disease 2019 (COVID-19) as of October 18, 2022 [1]. In response to a sudden surge in incidence during late March 2020 (first wave), the national government declared the first state of emergency, requesting residents to avoid unnecessary physical contact. The first state of emergency was declared on April 7, 2020 in seven (Saitama, Chiba, Tokyo, Kanagawa, Osaka, Hyogo, and Fukuoka) prefectures. That state of emergency was expanded to all 47 prefectures on April 16, 2020. The state of emergency was gradually lifted on May 14 and fully lifted in all prefectures by May 25, 2020. During the state of emergency, essential interventions included requesting a voluntary reduction in nonessential physical contact, self-restraint regarding movement across prefectural borders, and reduced operating hours for night-life establishments (e.g., bars and restaurants). The first wave was brought under control as a result of these containment efforts by the Japanese government [2]. However, canceling the intervention and reopening society led to a resurgence in COVID-19 incidence, resulting in the second pandemic wave from June 2020.

Although the public health and social measures during the first state of emergency greatly reduced the disease burden in Japan, they also caused considerable economic damage to the tourism sector as well as eating and drinking establishments. Therefore, to reactivate regional economic activities and assist those sectors most affected by COVID-19, the national government implemented the Go To Travel campaign on July 22, 2020. The campaign was designed to offer discounts for stays at hotels and *ryokan* (traditional inns found throughout the country, notably in hot-spring resorts) and to issue regional coupons that could be redeemed for eating purposes [3]. When the campaign began, the pandemic was undergoing an increase in Tokyo. Accordingly, residents in and travelers to Tokyo were excluded from the first of the two campaigns [4]. Nevertheless, once the second pandemic wave was brought under control, the government sought to promote travel to and from Tokyo; the city was then included in the second campaign, which began on October 1, 2020. In addition to involving Tokyo, the financial scale of the second campaign was clearly greater than the first. The first campaign simply provided a travel discount for accommodation; however, the second campaign also offered regional discount coupons that could be used in local restaurants and bars. The second campaign was specifically renamed the Go To Eat campaign [5]. As a result, it is estimated that the economic boost from the first campaign starting in July 2020 was 313 billion yen and that from the second campaign was 2.335 trillion yen [6]. However, a resurgence of COVID-19 in late October 2020 resulted in the third pandemic wave in Japan, and the campaign was discontinued in mid-December 2020 [7].

In general, the impact of human mobility on the spatial spread of directly transmitted diseases is widely recognized [8–10]. Regarding COVID-19 and travel, the Cabinet Secretariat of Japan has analyzed and publicly shared the importation risk index. This is estimated based on a simple product of the trans-prefectural migration rate per capita and the incidence of SARS-CoV-2 infection in the prefectures of origin [11]. Studies have analyzed the spatial patterns of COVID-19 spread in the United States, Brazil, and the United Kingdom [12–14] as well as human mobility in Japan during the early phase of the pandemic [15–17]. When the first campaign was about to start, the incidence exhibited an upward trend, and many prefectures expressed concern over the spread of transmission. For example, the governors of Tokyo and Okinawa Prefecture (the southernmost prefecture, which attracts many tourists) requested people to refrain from unnecessary movement across prefectural borders even when the campaign was beginning [18, 19]. The coincidental second pandemic wave was dominated by urban prefectures, such as Tokyo and Osaka, and was gradually brought under control in early August. However, the above problems during the first campaign led public health experts to revisit and analyze the impact of enhanced travel on the spatial dissemination of infection.

In an earlier study, we examined the time-dependent change in the incidence of COVID-19 during the first Go To Travel campaign [20]. Our analysis of the first campaign was somewhat unclear in that the second wave was brought under control during that campaign; however, there was an abrupt change in the pandemic wave following the second campaign, which involved larger subsidies. Thus, in the present study, we focused on the second Go To Travel campaign to examine the relationship between enhanced travel and geographic propagation of COVID-19 across Japan. To identify a possible cause-outcome relationship, we analyzed the proportion of prefectures that experiencedCOVID-19 epidemic above a certain threshold incidence level and implemented an interrupted time-series analysis (causal inference method used in COVID-19-related studies) [21–26].

## Methods

### Pandemic data

In Japan, COVID-19 has been designated a notifiable infectious disease according to the Infectious Disease Control Law [27]; cases diagnosed using a nucleic acid amplification test or antigen test are notified as confirmed COVID-19 cases. Confirmed cases are mandatorily reported to the government within 24 h of diagnosis. During the study period of the present investigation, all suspected patients were requested to quarantine for 14 days [28], and all underwent real-time reverse transcription polymerase chain reaction testing on days 7 and 14. As a result of these control measures, the cumulative incidence of COVID-19 by the end of 2020 was 235,700 cases, which was less than 0.2% of the national population.

In this study, we obtained the COVID-19 incidence reported from September 10 to November 9, 2020 from open data published by the Ministry of Health, Labour and Welfare [29]. The data were based on an online surveillance system, the Health Center Real-time Information-sharing System on COVID-19 [30].

We intentionally restricted our analyses to this time frame, taking into account the 30 days before and after the start of the second travel campaign (October 1, 2020). The time delay in reporting SARS-CoV-2 infection in Japan was approximately 9 days during the study period [31]. To obtain an epidemic curve as a function of the time of infection, we shifted the entire epidemic curve (originally drawn by reporting date) to the left for a fixed time delay of 9 days. The reporting involved weekend bias; therefore, we used a 7-day rolling average, and we counted the number of prefectures that underwent an incidence of three, five, and seven COVID-19 cases per 100,000 population per week. These values were specifically used because the pandemic situation in each prefecture was classified into four discrete stages during late 2020 (Additional file 2: Tables S1 and S2). Detection of 15 cases per 100,000 population per week led prefectural governments to declare a stage III pandemic and consider stringent public health and social measures that could involve restrictions on movement and other personal rights (Additional file 2: Tables S1 and S2) [32]. Stage IV involved 25 cases per 100,000 people. Even if prefectures could control the pandemic below stage III, they were advised to monitor the incidence. Stage II did not involve an explicit threshold value: each prefecture was advised to determine the value based on the local epidemiological situation. The World Health Organization guidance applies thresholds for the level of community transmission of 20, 50, and 150 COVID-19 cases per 100,000 people [33]. Following our exploratory analysis, we found that the number of newly reported cases per population in Japan during the study period was relatively low compared with other nations. Fewer than four prefectures exceeded the threshold of 10 per 100,000 people. Thus, in the present study, we employed slightly lower threshold levels of three, five, and seven cases per 100,000 population per week to capture local epidemic activity. Those lower threshold levels allowed us to determine more clearly how the situation changed with low levels of incidence.

We obtained the population estimates by prefecture from the Statistics Bureau of Japan [34] and the daily average temperature in each prefecture during the corresponding time period from the Japan Meteorological Agency [35]. As the representative value for temperature across all 47 prefectures, we applied the median value of daily average temperature from the data for each prefectural capital. During the study period, the proportion of positive cases among the total number of weekly tests remained below 10%, and there were no major changes (Additional file 2: Figs. S1 and S2). We did not standardize the number of newly infected cases because there were no significant differences in the consistency of reporting among prefectures. In the study period, B.1.1.284 and B.1.1.214 were the dominant SARS-CoV-2 lineages, but they were not determined to be variants of concern (Additional file 2: Fig. S3).

### Interrupted time-series model

Given the start date of the second campaign (October 1, 2020) and 9 days’ reporting delay, we set a campaign period in the interrupted time-series model from October 10 to November 9, 2020. The control period was 30 days before the second campaign (intervention) period. Through the corresponding time periods, we investigated the number of prefectures that had COVID-19 incidence in excess of the defined thresholds for weekly incidence (i.e., three, five, and seven cases per 100,000 population), *Y*_t_, which can be modeled as model 1:

1 $${Y}_{t}={\beta }_{0}+{\beta }_{1}T+{\beta }_{2}{X}_{t}+{\beta }_{3}\left(T-{T}_{i}\right){X}_{t},$$

where *T* is the time elapsed from the start of observation, *X*_t_ is the dichotomous variable representing the campaign state (0, pre-campaign; 1, post-campaign), and *T*_i_ is the time when the campaign started. ${\beta }_{0}$ is the parameter for the baseline level of the outcome, ${\beta }_{1}$ for increase in the outcome following the time-unit increase, ${\beta }_{2}$ change in the level of outcome immediately after the campaign, and ${\beta }_{3}$ the rate of increase following the campaign.

It has been reported that temperature may be associated with SARS-CoV-2 transmission [36, 37]; therefore, we developed an extended model (model 2) by incorporating temperature into model 1. Model 2 was as follows:

2 $${Y}_{t}={\beta }_{0}+{\beta }_{1}T+{\beta }_{2}{X}_{t}+{\beta }_{3}\left(T-{T}_{i}\right){X}_{t}+{\beta }_{4}Z,$$

where *Z* is the median value of the daily average temperature for Japan’s 47 prefectures.

We also examined a model without considering the immediate change in outcome after implementing the second campaign (${\beta }_{2}$). Model 3 was as follows:

3 $${Y}_{t}={\beta }_{0}+{\beta }_{1}T+{\beta }_{3}\left(T-{T}_{i}\right){X}_{t}+{\beta }_{4}Z.$$

Assuming that ${Y}_{t}$ follows a Poisson distribution, we applied the maximum-likelihood method to estimate all parameters; we derived the 95% confidence intervals (CIs) of the estimates using the parametric bootstrap method with 10,000 samples. Lastly, to select the best model among those proposed, we calculated the Akaike information criterion.

To examine the robustness of our results, we conducted sensitivity analyses. Our sensitivity analysis covered different geographic groups, timing of the intervention, holiday periods, the different models, and the study period (additional analysis). To exclude the possibility that an increase in infections was caused simply by geographic bias—especially heterogeneities associated with urbanization—we conducted a subgroup analysis using two discrete prefectural groups (urban and nonurban groups) based on each prefecture’s population density. To account for uncertainty about the time of illness onset to official reporting, we undertook sensitivity analysis using different values for the timing of the intervention. We also conducted interrupted time-series analyses: (1) using holiday periods as a possible explanatory variable; (2) employing another functional model with an exponential function for inference; and (3) extending the length of the study period until termination of the campaign.

### Data-sharing statement

The original data analyzed in the present study are available in the Additional file 1.

## Results

**Fig. 1:**
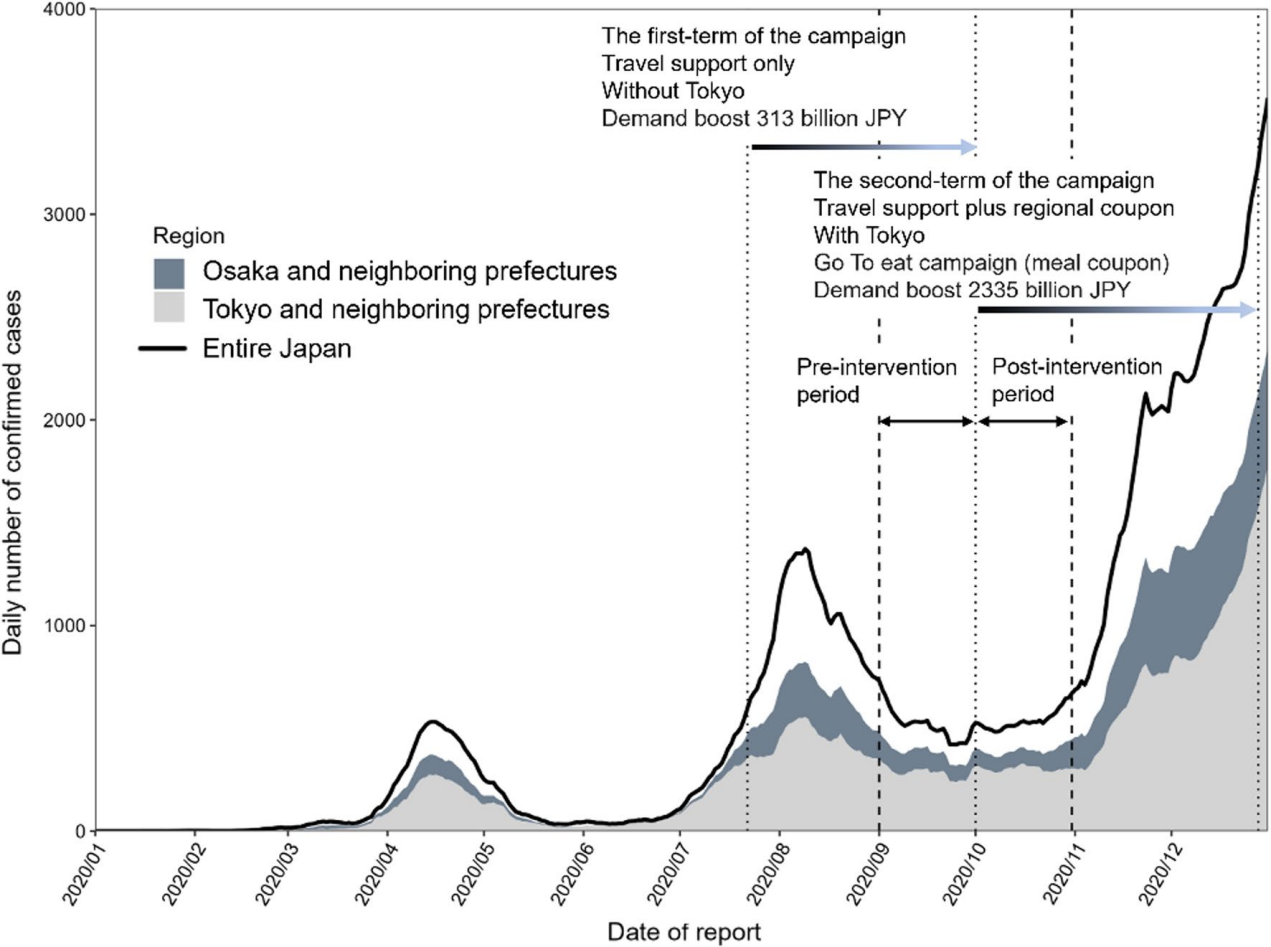
Epidemic curve for confirmed cases of COVID-19 in Japan, 2020. The number of cases (7-day moving average) throughout Japan (solid line) is shown. The fractions in Osaka and neighboring prefectures (dark gray) and Tokyo and neighboring prefectures (light gray) are indicated separately. The neighboring prefectures of Osaka comprise Kyoto, Nara, and Hyogo; those of Tokyo comprise Kanagawa, Saitama, Chiba, and Yamanashi. The first campaign began on July 22, 2020 and the second on October 1, 2020. The study period was September 1 to October 31, 2020

Figure 1 shows the pandemic curve of confirmed COVID-19 cases in 2020; it highlights the contribution of urban prefectures, especially Tokyo, Osaka, and neighboring prefectures. By the end of 2020, there were 234,109 COVID-19 cases diagnosed, of which 154,380 (66%) were in the above prefectures. Before the start of the second Go To Travel campaign, 57,076 cases (69%) were detected in Tokyo, Osaka, and neighboring prefectures.

**Fig. 2:**
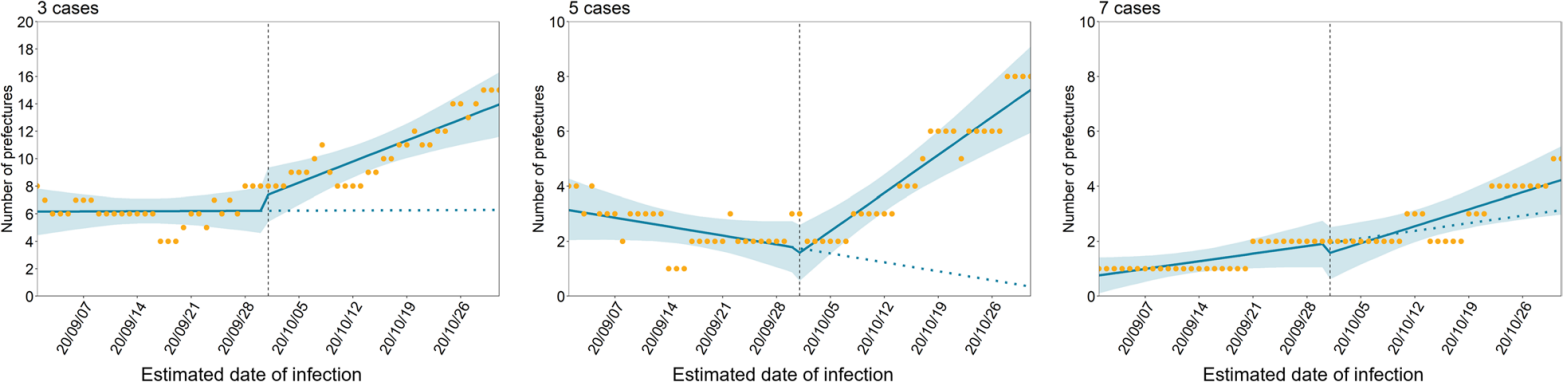
Effect of Go To Travel campaign on number of prefectures exceeding certain incidence criteria. We counted the prefectures where the number of newly reported cases (per 100,000 population) before and after the campaign (intervention) exceeded the threshold values of three, five, and seven cases per week. The vertical lines indicate when the second campaign (intervention) started. The yellow dots represent observed data; the blue lines show the estimated results of the interrupted time-series analysis. The blue shaded areas indicate 95% confidence intervals for the modeled results. The blue dashed lines during the second campaign period represent the estimated counterfactual scenario, i.e., if the campaign had not been launched. Model 1 assumed that there were changes in the baseline level and rate of increase following the second campaign

Figure 2 presents a comparison between the observed and modeled number of prefectures where COVID-19 incidence exceeded the thresholds defined for analysis (three, five, and seven cases per 100,000 population per week) based on model 1. Clear increasing trends in the number of prefectures above all thresholds were evident after implementation of the second campaign. Examination of those trends shows a significant increase in ${\beta }_{3}$, representing the rate of increase following the second campaign (Table 1). ${\beta }_{3}$ was estimated to be 0.22 (95% CI 0.062 to 0.37) and 0.24 (95% CI 0.15 to 0.34) at thresholds of three and five cases, respectively. However, for all thresholds, the 95% CIs for ${\beta }_{1}$ and ${\beta }_{2}$, which indicated the increase in the outcome following the time-unit increase and changes in the outcome immediately after the second campaign, included zero. We obtained similar results in the subgroup analysis, which divided prefectures into two groups (i.e., the top 25% prefectures with high population density and others) (Additional file 2: Fig. S4). Changes in the slope before and after the campaign were also evident in the analyses when the start date for the campaign was shifted (± 5 days from the original date) (Additional file 2: Fig. S5).

**Table 1:** Estimates for the level and slope change in model 1

	3 cases/100,000 population/week	5 cases/100,000 population/week	7 cases/100,000 population/week
${\beta }_{0}$ (intercept)	6.17 (4.50, 7.85)	3.14 (2.00, 4.27)	0.76 (0.10, 1.40)
${\beta }_{1}$ (linear trend)	0.0020 (− 0.097, 0.099)	− 0.047 (− 0.11, 0.014)	0.040 (− 0.0048, 0.083)
${\beta }_{2}$ (intercept change following campaign)	1.16 (− 1.49, 3.80)	− 0.16 (− 1.58, 1.27)	− 0.37 (− 1.66, 0.93)
${\beta }_{3}$ (change in the rate of increase following campaign)	0.22 (0.062, 0.37)	0.24 (0.15, 0.34)	0.048 (− 0.027, 0.13)

Numbers in parentheses represent 95% confidence intervals calculated using the bootstrapping method

**Fig. 3:**
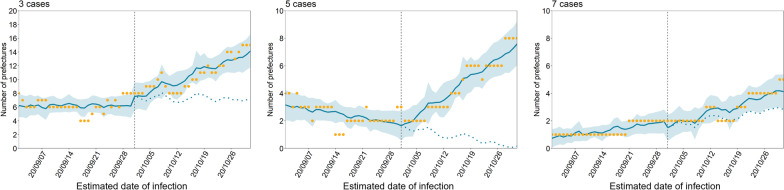
Effect of Go To Travel campaign on prefectures exceeding certain criteria in Model 2. The number of prefectures where the number of newly reported cases (per 100,000 population) before and after the campaign (intervention) exceeded the threshold values of three, five, and seven cases per week are shown. The vertical lines indicate when the second Go To Travel campaign (intervention) started. The yellow dots represent observed data; the blue lines show the estimated results of the interrupted time-series analysis. The blue shaded areas indicate 95% confidence intervals for the modeled results. The blue dashed lines during the second campaign period represent the estimated counterfactual scenario, i.e., if the campaign had not been launched. Compared with model 1, which assumed changes before and after the second campaign, model 2 additionally accounted for temperature

Figure 3 shows the results based on model 2, which addressed temperature dependence. Even after adjusting for temperature, the rate of increase in the number of prefectures with the defined threshold number of cases per 100,000 population was clearly evident. In addition to temperature, the impact of holiday periods was also considered in the analysis: there were no significant differences in the results for model 1 (Additional file 2: Fig. S6).

**Fig. 4:**
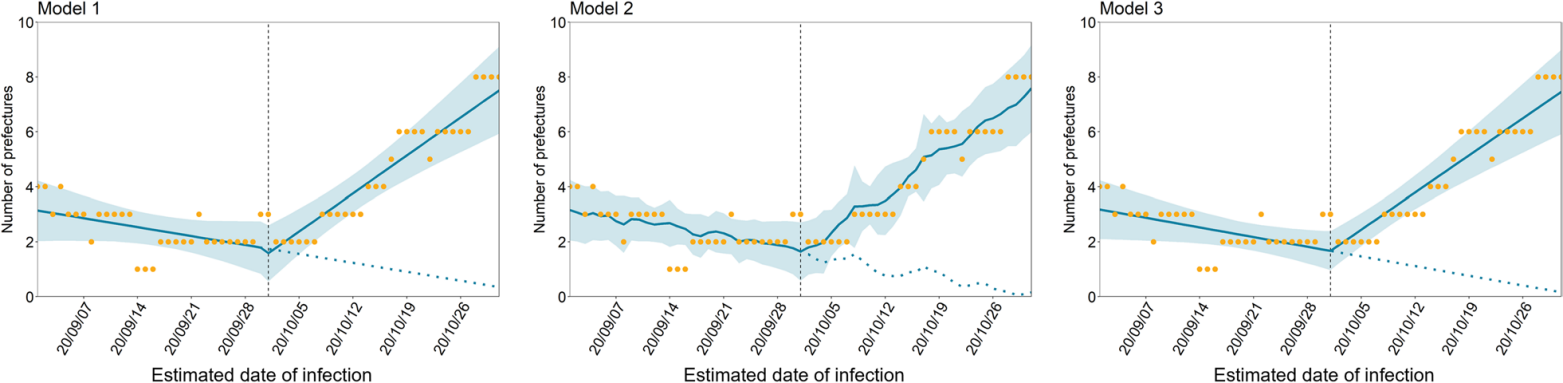
Effect of Go To Travel campaign prefectures above threshold values for incidence across different models. We counted the prefectures where the number of newly reported cases before and after the campaign (intervention) exceeded five cases per 100,000 population per week. The vertical lines indicate when the second campaign (intervention) started. The yellow dots represent observed data; the blue lines show the estimated results of the interrupted time-series analysis. The blue shaded areas indicate 95% confidence intervals. The blue dashed lines during the second campaign period represent the estimated counterfactual scenario, i.e., if the campaign had not been launched. Model 1 assumed changes in the baseline level and rate of increase following the second campaign. Model 2 accounted for temperature in addition to the causal effect of the campaign. Compared with model 1, model 3 assumed that only the change in the rate of increase occurred following the campaign

In Fig. 4, we compare results from the proposed models when the threshold was fixed at the number of prefectures with five or more cases per 100,000 population. In all analyses with the three different models, ${\beta }_{3}$, measuring the rate of increase in the number of prefectures, always had a positive value, including a lower 95% CI (Table 2). There was a consistent rise in the rate of increase in the number of prefectures after the second campaign—both with and without taking temperature into account and with and without the baseline change immediately after the campaign (${\beta }_{2}$). Comparing Akaike information criterion values, model 3, which accounts for the rate of change but not change in the baseline level through the campaign, was selected as the best model, yielding the minimum value of 199.9. When we extended the study period and involved two change points (the start and end dates of the campaign), we also obtained generally comparable results (Additional file 2: Figs. S7 and S8). However, population-based interventions were in place at the end of campaign, and so mobility could also have been altered by the interventions.

**Table 2:** Comparison of estimates for the level and slope change in each model

	Model 1	Model 2	Model 3
${\beta }_{0}$ (intercept)	3.14 (2.00, 4.27)	5.78 (− 4.88, 16.25)	3.18 (2.13, 4.24)
${\beta }_{1}$ (linear trend)	− 0.047 (− 0.11, 0.014)	− 0.073 (− 0.19, 0.048)	− 0.050 (− 0.099, − 0.0011)
${\beta }_{2}$ (intercept change following campaign)	− 0.16 (− 1.58, 1.27)	− 0.042 (− 1.56, 1.47)	–
${\beta }_{3}$ (change in the rate of increase following campaign)	0.24 (0.15, 0.34)	0.25 (0.15, 0.34)	0.24 (0.15, 0.34)
${\beta }_{4}$ (coefficient for temperature)	–	− 0.092 (− 0.45, 0.28)	–

Numbers in parentheses represent 95% confidence intervals calculated using the bootstrapping method

## Discussion

To establish whether the second Go To Travel campaign accelerated the geographic spread of COVID-19 in Japan, we conducted an interrupted times-series analysis of the number of prefectures that exceeded a defined threshold number of cases per 100,000 population. Three threshold values and three different models identified an acceleration in the rate of increase in the number of prefectures above the threshold: they indicated that the inter-prefectural spread of COVID-19—especially from urban to nonurban areas—was intensified following the launch of the second campaign. Our analysis did not exclude other factors that could have contributed to the increased spread of COVID-19. However, considering the plausibility of alternative explanations (discussed below), we believe that promoting travel resulted in accelerating the inter-prefectural spread of infection.

An important finding is that several nonurban areas became affected by COVID-19 after acceleration of the urban-to-nonurban spread following the second Go To Travel campaign, which allowed travel to and from Tokyo. During the pre-campaign period (during the first Go To Travel period), the rate of increase in the number of prefectures that exceeded the defined thresholds ranged from − 0.047 to 0.040 per day, i.e., a negative or small positive value; however, that increased to 0.048 to 0.24 per day during the post-campaign period. Even when we used a different model (e.g., an exponential model instead of a linear model to capture the time-dependent trend), we obtained similar results with consistent interpretations (Additional file 2: Fig. S7).

It should be noted that the Go To Travel campaign was a crucial policy for assisting the tourism, transport, and restaurant sectors after the sharp reduction in economic activity associated with COVID-19 interventions. However, the policy spread the pandemic to nonurban prefectures that did not possess sufficient health-care capacity to meet the hospital case load demand [38]. That effect could have been reduced with a greater proportion of immunized individuals through either vaccination or natural infection. However, given the immune escape potential of new SARS-CoV-2 variants, the substantial risk of geographic spread owing to travel campaigns would not be negligible should a similar travel campaign be introduced while the incidence level remains high.

We incorporated temperature into our analysis because our earlier study and others indicated that decreased temperature would have an impact on secondary SARS-CoV-2 transmission [31, 39–41]. The Go To Travel campaign took place from summer to winter, with gradually lowering temperatures. We estimated the temperature coefficient, ${\beta }_{4}$, to be − 0.092 (95% CI − 0.45 to 0.28), which was not statistically significant. However, even after adjusting for the potential influence of temperature, the effect of the second campaign remained evident.

In this research, we investigated the geographic spread of COVID-19 infection rather than examining the transmission dynamics within each prefecture. During the study period, the SARS-CoV-2 lineages B.1.1.284 and B.1.1.214 were dominant. Neither of them has been classified as a variant of concern, but they share a point mutation (D614G) in the viral spike protein. That may have affected the transmission dynamics; however, we did not have access to genomic surveillance by geographic region, and it was very difficult to judge whether those lineages affected the spatial spread of COVID-19 (Additional file 2: Fig. S3). It is highly likely that changes in inter-prefectural movement correlated with implementation of the campaign; thus, in the regression model, we considered only temperature without a potentially correlated value.

In this study, we did not account for changes in the spread of COVID-19 in each prefecture because the pandemic activity in the affected prefectures remained small. Thus, to determine the causal impact of Go To Travel using empirically observed epidemiological data, we considered that analyzing the initiation of the pandemic would be the most measurable outcome. Our purpose was to examine the inter-prefectural spread of COVID-19 before and after the travel campaign—not changes in the number of newly infected cases within a prefecture. Therefore, we focused on the number of prefectures that exceeded the threshold values rather than directly determining the incidence level and decomposing the transmission dynamics in detail. To compare the level of actual pandemic magnitude by prefecture, it would perhaps be necessary to account for different interventions adopted in each prefecture [42].

Our interrupted time-series analysis model examined abrupt changes by comparing the number of prefectures with a certain level of incidence before and after the second travel campaign; however, it is useful to explore whether the outcome also changed (e.g., decreased) after cancellation of the campaign. We examined whether results that were consistent with what we obtained in the primary investigation were maintained after accounting for the post-campaign period (Additional file 2: Fig. S8). It was very difficult, however, to explicitly assess the impact of suspending the campaign using the interrupted time-series analysis model. The Go To Travel campaign was gradually suspended from late November 2020 in prefectures where the number of people with COVID-19 increased significantly; the program was suspended nationwide on December 28, 2020. On January 7, 2021, a state of emergency was declared for Tokyo and surrounding areas. Considering the overlapped timing between cessation of the campaign and implementing public health and social measures, we believe that as well as suspending the campaign the impact of various countermeasures and people’s risk awareness affected the subsequent decline in the outcome. It was difficult to assess the impact of campaign suspension because the timing of the campaign’s end differed by prefecture; travel-associated cases represented only a small fraction of COVID-19 cases when the number of infected people substantially increased (Additional file 2: Fig. S8).

This study has some limitations. The first and most important is that the causal argument based on interrupted time-series analysis is vulnerable to concurrent changes that happened at a similar time to the campaign. A plausible alternative explanation cannot be identified, but the second campaign took place during a period when the following occurred: (1) restrictions on mass gatherings were eased [43]; (2) entrance from overseas to Japan was partially allowed for reentry visa holders [44]; and (3) onsite school classes restarted. Nevertheless, it is unlikely that these directly influenced the spatial spread of COVID-19 in Japan, and the involvement of schoolchildren in transmission remained minimal during the latter part of 2020 [45].

The second limitation is associated with ascertainment bias. The Go To Travel campaign featured in the mass media during the second campaign period, and the pandemic in Tokyo then was increasing. Thus, recognizing the pandemic risk may have been heightened, and later cases may have been better identified than in earlier periods. However, even with improved case detection, it is difficult to explain the steady increase in the number of prefectures with a greater number of cases during the campaign period.

Third, we focused on the second Go To Travel campaign, and the control period was during the latter period of the first campaign. The first campaign had limited impact on the geographic spread of COVID-19. During the first campaign, the second wave in Japan (which was dominated by urban prefectures, including Tokyo and Osaka) was gradually brought under control, and the number of travel-associated cases did not increase. Moreover, the budget of the first Go To Travel campaign was limited compared with that of the second campaign [46, 47]. Thus, we considered the first campaign period an appropriate control period to measure the impact of the second campaign.

Fourth, we did not consider inter-prefecture mobility rate owing to scarcity of data. Currently available human movement data are restricted to mobility (and de facto population) within each prefecture, not the actual rate of movement crossing prefectural borders.

## Conclusions

We believe the enhanced travel resulting from the Go To Travel campaign facilitated inter-prefectural spread of COVID-19. Policies that boost economic activities should be balanced with the increase in pandemic risk. It must be remembered that promoting travel may cause an epidemic to be easily disseminated from urban to nonurban locations, where health-care capacity may be limited.

## Supplementary Information

**Additional file 1.** Number of prefectures that exceeded the threshold number of cases per 100,000 persons.

**Additional file 2.** Supplementary text containing supplementary Tables S1 and S2 and supplementary Figures S1–S8.

## Data Availability

The original data analyzed in the present study are available in the Additional file 1.

## Abbreviations

COVID-19: Coronavirus disease 2019
SARS-CoV-2: Severe acute respiratory syndrome coronavirus 2
