# Supplementary material for: Go To Travel campaign and the geographic spread of COVID-19 in Japan
Source: BMC Infect Dis. 2022 Oct 31;22:808. doi: 10.1186/s12879-022-07799-0 (PMC9619015; doi:10.1186/s12879-022-07799-0)
Supplement: Supplementary file 2 — Additional file 2. Supplementary text containing supplementary Tables S1 and S2 and supplementary Figures S1–S8. [file 12879_2022_7799_MOESM2_ESM.docx]

**Additional Table S1.** Four stages of the COVID-19 epidemic in Japan

| Stage | Situation | Interventions |
| --- | --- | --- |
| Stage IV | Stage in which action is needed to avoid the explosive spread of infection and severe failure of the health care provision system | Reduction in overall contact opportunities  - Declaration of a State of Emergency  - Request to refrain from traveling to other prefectures  - School closure |
| Stage III | Stage in which action is needed to avoid a rapid increase in the number of cases and significant problems in the health care provision system | Reduction of contact opportunities depending on the situation  - requested shortened business hours  - Request for high-risk groups to refrain from traveling to the areas where the infection has spread  - Restrictions on the number of patrons allowed in the restaurant |
| Stage II | Stage of gradual increase in the number of cases and accumulation of burden on the health care provision system | Basic infection prevention measures  Early detection and response to clusters  Risk assessment for control of infection |
| Stage I | Sporadic occurrence of cases and no problem in the medical care provision system | Basic infection prevention measures  Early detection and response to clusters  Risk assessment for control of infection |

(Modified from source: Cabinet Secretariat. 2020. [1])

**Supplementary Table S2.** Indicators of the four stages of the COVID-19 epidemic in Japan

|  | Medical Care Provision System | | | Surveillance system | Epidemiological Situation | | |
| --- | --- | --- | --- | --- | --- | --- | --- |
|  |  | | Active cases | PCR positivity rate | Number of newly cases | Compared to last week | Proportion of unknown transmission route |
|  | Number of beds | Number of beds for severe cases |  |  |  |  |  |
| Stage IV | More than 1/2 of maximum beds secured | More than 1/2 of maximum beds secured | 25 cases/ 100,000 population | 10% | 25 cases/ 100,000 population/ week | >1 | 50% |
| Stage III | More than 1/5 of maximum beds secured | More than 1/5 of maximum beds secured | 15 cases/ 100,000 population | 10% | 15 cases/ 100,000 population/ week | >1 | 50% |
| Stage II | - | - | - | - | - | - | - |
| Stage I | - | - | - | - | - | - | - |

PCR, polymerase chain reaction.

(Modified from source: Cabinet Secretariat. 2020. [1])

Supplementary material

**A. Proportion of positive cases of SARS-CoV2 infection by prefecture before and after the Go To Travel campaign began**

To verify that there were no significant differences in ascertainment bias between prefectures, the figures for the proportion of positive COVID-19 cases per prefecture before and after the start of the campaign and over time are shown. The data on the proportion of positives are published by the Ministry of Health, Labour and Welfare [2]. These datasets not only include testing for diagnosis but also for discharge.


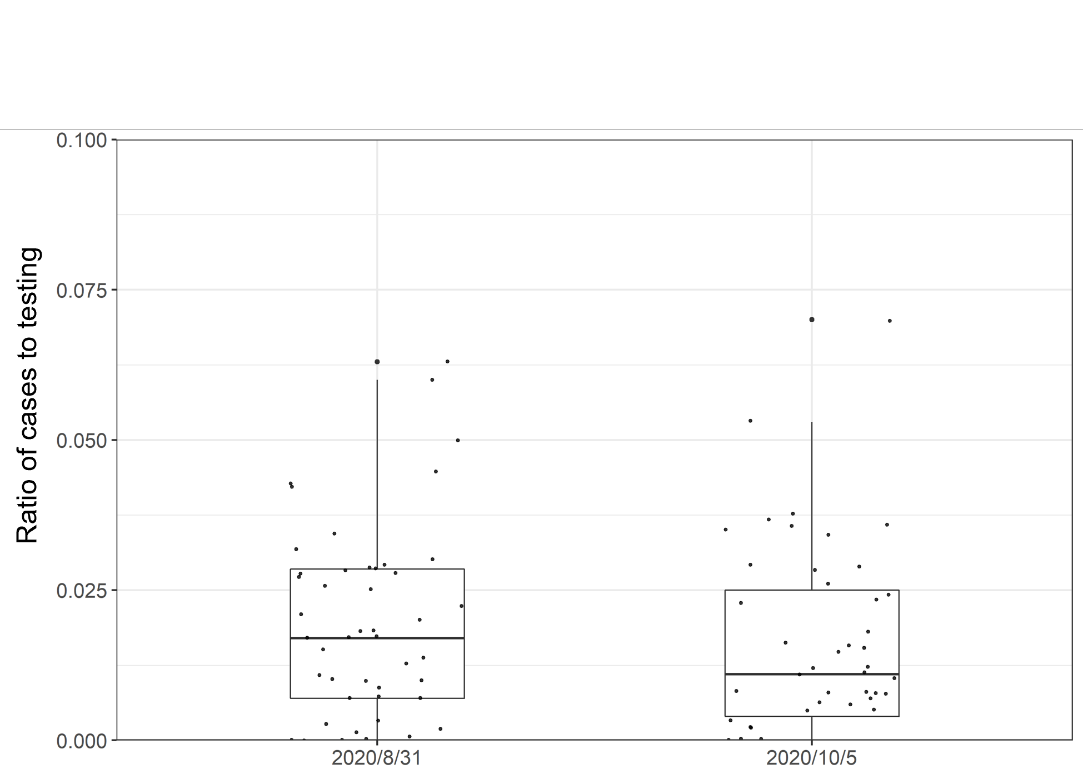


**Supplementary Figure S1. Proportion of positive COVID-19 cases by prefecture before and after the Go To Travel campaign.** The proportion of positive cases by prefecture is shown. Each dot represents a prefecture. Data are shown immediately before the start of the study period (31 August 2020) and immediately after the start of the campaign on 1 October 2020 (5 October 2020 was selected as it was the first Monday of the campaign and the original data were collected on a weekly basis). The boxes range from the 25th to 75th percentile, and the whiskers indicate 1.5 times the interquartile range.


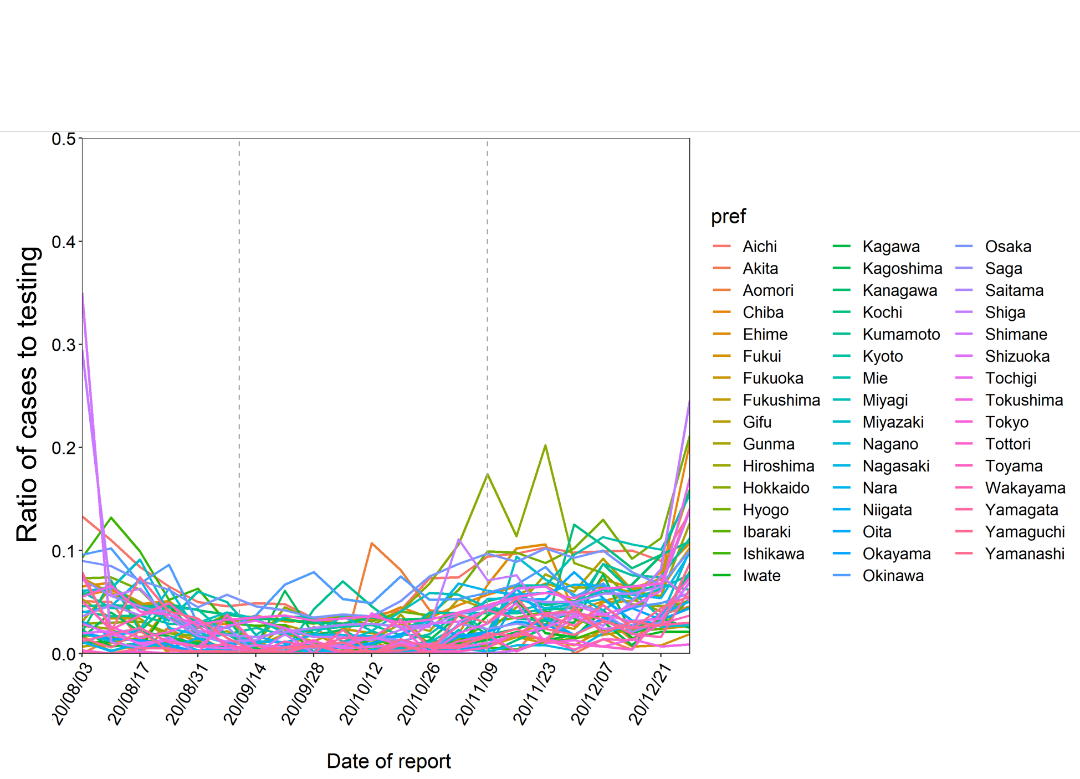


**Supplementary Figure S2. Trend in the proportion of positive COVID-19 cases by prefecture before and after the campaign.** Weekly data on the proportion of positive cases by prefecture are shown. The two vertical dashed lines indicate the beginning and end of the study period, respectively.

**B. Genomic surveillance in Japan and the number of prefectures exceeding the threshold**

Genomic surveillance results [3] and number of prefectures are shown, confirming the impact of SARS-CoV-2 variants.


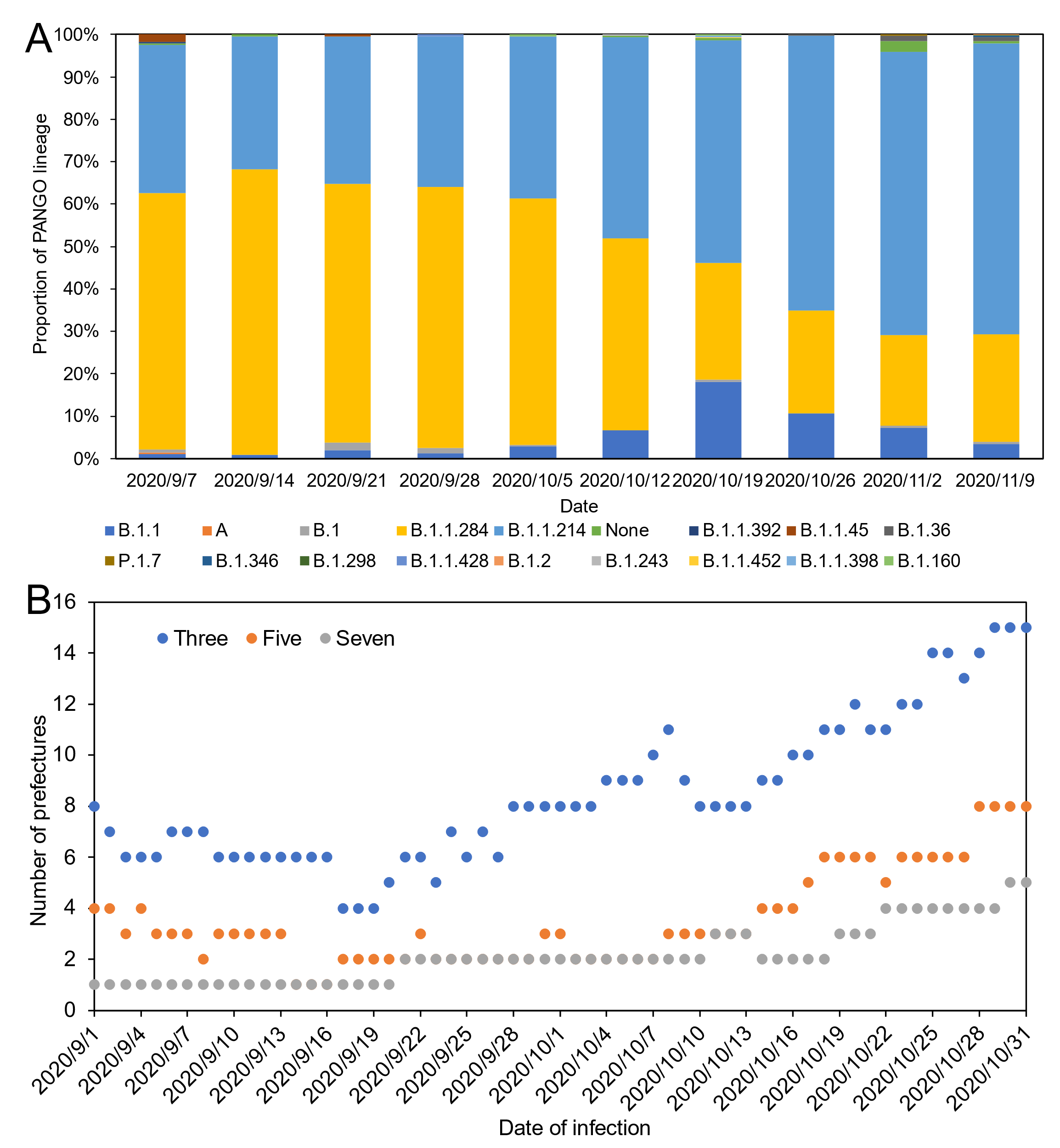


**Supplementary Figure S3. Genomic surveillance of SARS-CoV-2 in Japan and the number of prefectures exceeding the threshold.** Weekly data on the genomic surveillance of SARS-CoV-2 and the number of prefectures where the number of newly reported cases exceeded certain thresholds are shown.

PANGO, Phylogenetic Assignment of Named Global Outbreak.

**C. Sub-analysis after dividing prefectures into two groups according to population density**

To exclude the possibility that the increase was simply caused by geographic bias, especially the influence of urban areas, subgroup analysis was performed using two prefectural groups based on population density [4]. Urban prefectures were defined as those with the top 25% of population density among densely inhabited districts. The analysis was performed using a threshold of 3 cases per 100,000.


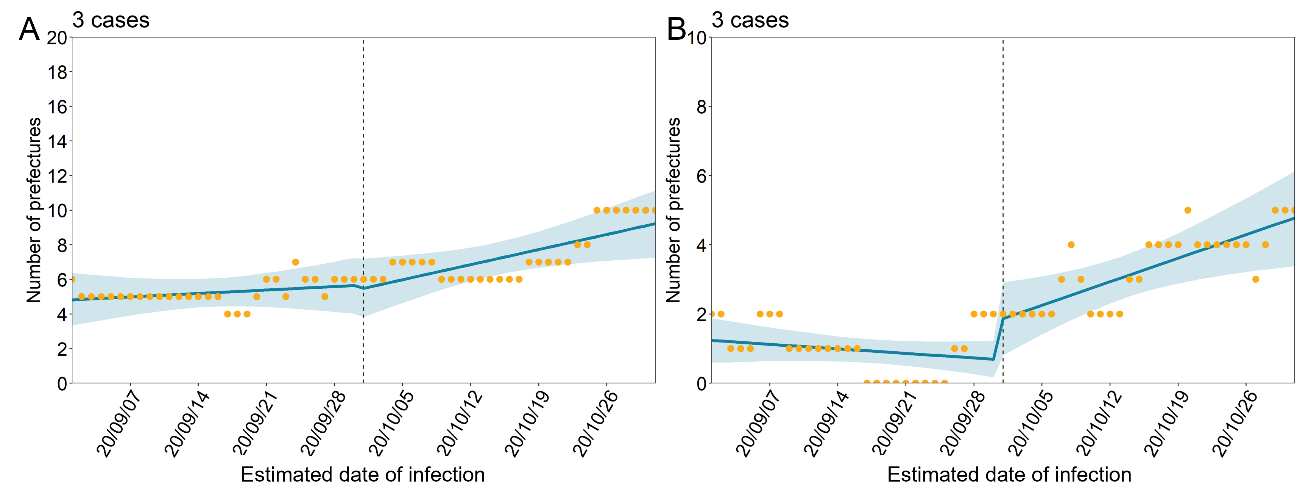
 **Supplementary Figure S4. Sub-analysis of Go To Travel campaign effect on prefectures exceeding certain criteria for two groups.** The number of prefectures where the number of newly reported cases before and after the campaign exceeded the threshold of 3 cases/100,000 population/week is shown. Dots represent observed data, lines represent the estimated results via interrupted time-series analysis, and shaded areas indicate 95% confidence intervals. Panel A shows the results when only urban prefectures are used, and panel B shows the results when prefectures other than the top 25% of urban locations are used.

**D. Sensitivity analysis by shifting the timing of second campaign (intervention)**

In the present study, the time from illness onset to reporting was fixed. Results using different values (considering uncertainty) are shown in Figure S5.


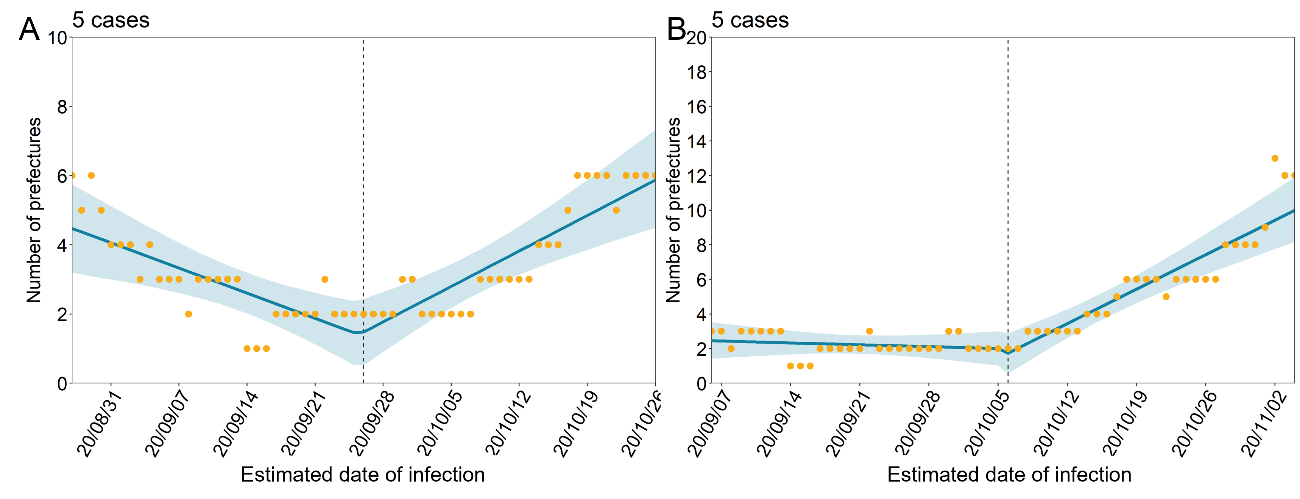
 **Supplementary Figure S5. Sensitivity analysis of Go To Travel campaign effect on prefectures exceeding criteria, shifting intervention timing.** The number of prefectures where the number of newly reported cases before and after the campaign exceeded the threshold of 5 cases/100,000 population/week are shown. Dots represent observed data, lines represent the estimated results via interrupted time-series analysis, and shaded areas indicate 95% confidence intervals. Panel A shows the results of shifting the timing of campaign by minus 5 days, and panel B shows the results of shifting the timing by plus 5 days.

**E. Sensitivity analysis including holiday periods**

As the present study included only temperature as an adjustment, the results using holiday periods as another possible factor are shown in Supplementary Figure S6. Both the holiday periods 19 September to 22 September and 19 September to 27 September were considered.


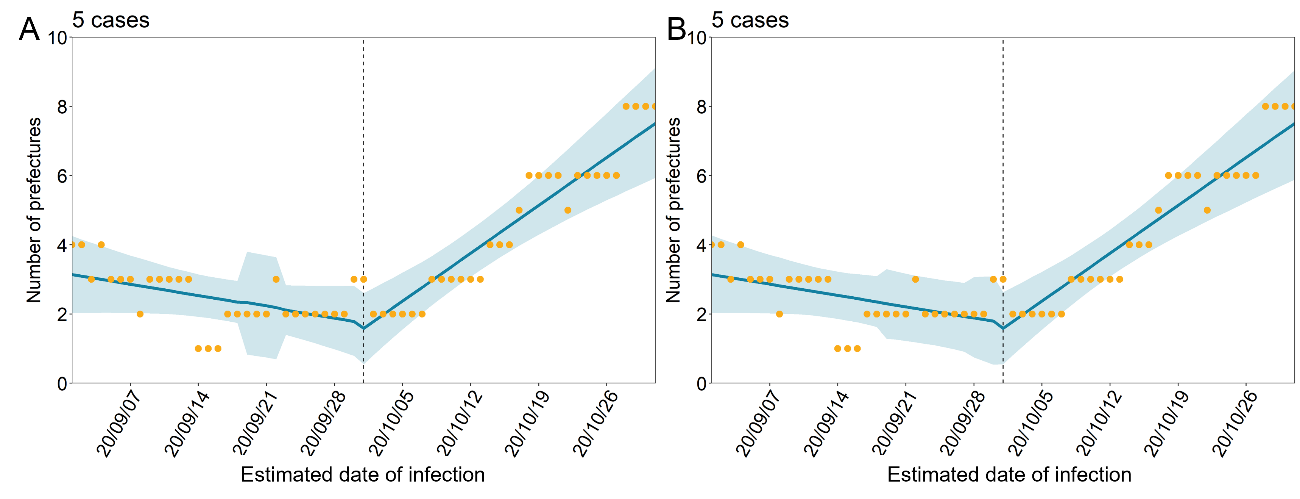
 **Supplementary Figure S6. Sensitivity analysis of Go To Travel campaign effect on prefectures exceeding certain criteria including holidays.** The number of prefectures where the number of newly reported cases before and after the campaign exceeded the threshold of 5 cases/100,000 population/week are shown. Dots represent observed data, lines represent the estimated results in interrupted time-series analysis, and shaded areas indicate 95% confidence intervals. Panels A and B show the results when 4 days and 7 days of the holiday periods were considered respectively.

**F. Sensitivity analysis using an exponential model**


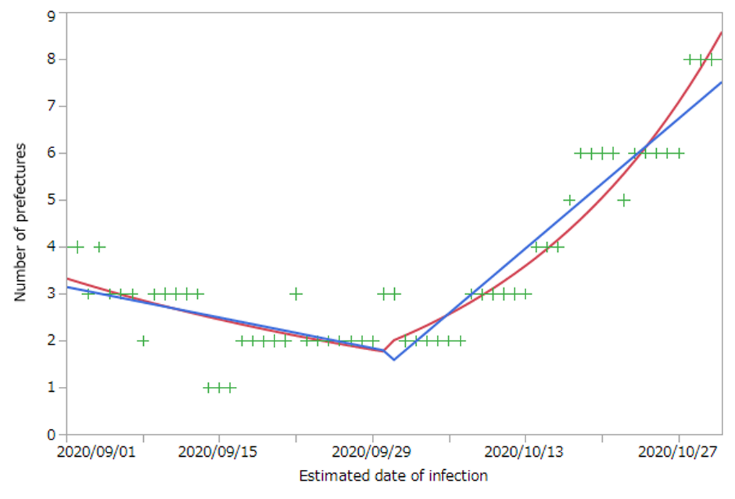
In the present study, interrupted time-series analysis was conducted using a linear model. Results using an exponentioal model are shown in Figure S7.

**Supplementary Figure S7. Sensitivity analysis of Go To Travel campaign effect on prefectures exceeding criteria using exponential model.** The number of prefectures where the number of newly reported cases before and after the campaign exceeded the threshold of 5 cases/100,000 population/week are shown. Dots represent observed data, blue lines represent the estimated results in interrupted time-series analysis using the original model, red lines represent the estimated results in interrupted time-series analysis using an exponential model, and shaded areas indicate 95% confidence intervals.

**G. Sub-analysis by extending the study period with two change points**

The present study only considered one intervention, the start of the Go To Travel campaign. Figure S8 shows the results when a second campaign, suspending the campaign, is considered.
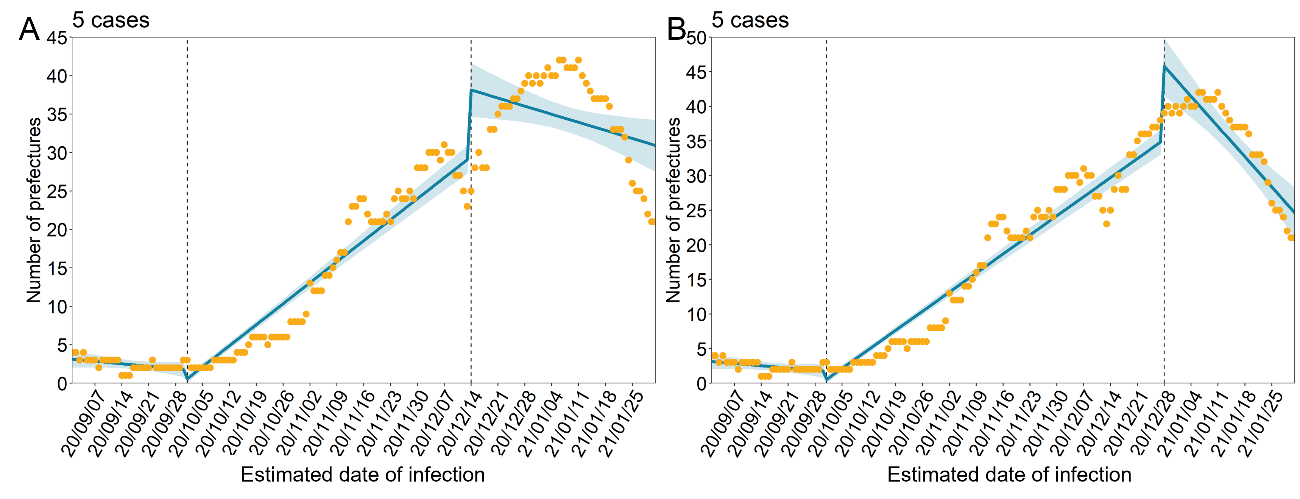


**Supplementary Figure S8. Sub-analysis of Go To Travel campaign effect on number of prefectures exceeding certain criteria.** The number of prefectures where the number of newly reported cases before and after the campaign exceeded the threshold of 5 cases/100,000 population/week are shown. Dots represent observed data, lines represent the estimated results with interrupted time-series analysis, and shaded areas indicate 95% confidence intervals. Panels A and B show the results for the second change points of 14 and 28 December, respectively.

**References**

1. Cabinet Secretariat. Indicators for the implementation of countermeasures in response to changes in the infection situation. 2020. https://corona.go.jp/news/pdf/jimurenraku_0811.pdf. Accessed 7 Sep 2022.

2. Ministry of Health, Labour and Welfare. COVID-19 Advisory Board. 2021. https://www.mhlw.go.jp/content/10900000/000731936.pdf. Accessed 4 Sep 2022.

3. National Institute of Infectious Diseases. Genomic surveillance of SARS-CoV-2. 2021. https://www.niid.go.jp/niid/ja/from-lab/2521-cepr/11261-cepr-topics-log.html. Accessed 4 Sep 2022.

4. Ministry of Internal Affairs and Communications. 2020 Population Census. 2021. https://www.stat.go.jp/english/data/kokusei/index.html. Accessed 4 Sep 2022.
